# Resilience and adaptive strategies for managing stigma in Chinese people with chronic kidney disease

**DOI:** 10.1038/s41598-025-12676-2

**Published:** 2025-07-23

**Authors:** Wei Xiong, Tianhui An

**Affiliations:** 1 School of Chinese Language and Literature, Central China Normal University, Wuhan, Hubei China; 2 Department of Geriatrics, Union Hospital, Tongji Medical College, Huazhong University of Science and Technology, Wuhan, Hubei China

**Keywords:** Chronic kidney disease, Stigma, Coping strategies, Qualitative research, China, Health occupations, Medical research, Nephrology

## Abstract

Chronic kidney disease (CKD) is a significant global public health issue, with a substantial population in China. Influenced by traditional Chinese medicine that links kidney health to sexual vitality, people with CKD in China face unique societal stigmatization. This study aimed to explore the stigmatization experiences of individuals with CKD in China and their corresponding coping strategies. Employing a focused ethnographic approach, this research utilized participant observation within three WeChat groups and conducted offline semi-structured interviews with 30 individuals. The analysis of interviews and field transcripts reveals that people with CKD in China adopt a dualistic coping strategy metaphorically described as “huddling for warmth”: they externally conceal their condition to avoid social stigma while internally cultivating a strong communal identity through online and offline interactions. Despite the prevalent stigma, these individuals demonstrate remarkable resilience and agency through their proactive coping mechanisms. This study highlights the urgent need for the Chinese government, medical institutions, and the public to address this deep-rooted stigma and foster a more inclusive and supportive environment to enhance the well-being of people living with CKD.

**Supplementary Information:**

The online version contains supplementary material available at 10.1038/s41598-025-12676-2.

Chronic kidney disease (CKD) impacts over 10% of the global population, amounting to more than 800 million individuals^1^making it a significant public health challenge^2^. This burden intensifies as the disease progresses through its clinical stages (G1-G5), often culminating in kidney failure (also known as end-stage renal disease, ESRD)^3^. At this advanced stage, individuals require life-sustaining kidney replacement therapy (KRT), such as dialysis or transplantation.

Beyond its clinical manifestations, CKD profoundly impacts the daily lives of those affected, imposing rigorous dietary restrictions, chronic fatigue, and a significant psychological burden that diminishes quality of life^4,5^. In managing this long-term condition, the unique, supportive relationships that often form between individuals with CKD and their healthcare providers, particularly renal nurses, can serve as a crucial buffer against daily challenges^6,7^. At the same time, the entire spectrum of CKD, and particularly the need for KRT, carries a heavy economic cost for both individuals and their families^8^contributes to national productivity losses^9^and places immense pressure on global healthcare systems^10,11^.

Furthermore, CKD is positively correlated with health disparities, exhibiting a clear social gradient in its prevalence. Factors such as gender^12,13^ race/ethnicity^14,15^, religion/ spirituality^16,17^, immigrant^18,19^ education^20^ socio-economic status^21^ occupation^22,23^ and place of residence^24,25^ significantly influence an equity-focused assessment of CKD. The growing population of people with CKD and the consequent strain on health systems necessitate a unified and coordinated response to CKD across both developed and developing countries to prevent a major health catastrophe^26,27^. Addressing this requires attention to the specific political, economic, and socio-cultural contexts of people’s lives, making qualitative research an essential tool^28^. The number of qualitative studies on people with CKD is gradually increasing, with a focus on their disease experiences^29^ perceptions^30^ and the impact of these factors on treatment and care^31^ as well as healthcare professionals’ views on treatment decisions^32,33^. However, qualitative research on people with CKD in China remains limited.

This research gap is particularly critical given the scale of the challenge in China. According to available data, China currently has 159.8 million people with CKD, making up 11.4% of its population and 20% of the world’s total^34^. Moreover, the CKD landscape in China is shifting towards patterns seen in developed countries. This transition is poised to significantly affect both China’s healthcare system and the global CKD landscape^35^. Influenced by traditional Chinese medicine that links kidney health to sexual vitality, people with CKD in China face unique societal stigmatization. Therefore, this study aimed to explore the stigmatization experiences of individuals with CKD in China and their corresponding coping strategies. By observing their daily lives, this research illuminates the socio-cultural stigmatization they face, thus underscoring their resilience and agency.

## Methods

### Study design

We conducted focused ethnographic research, including participant observation and semi-structured interviews. Focused ethnography merges inductive, immersive, and multimodal ethnographic research methods in a qualitative inquiry^36^. The research methodology follows the COREQ (Consolidated Standards of Reporting Qualitative Research) guidelines^37^.

### Participants and recruitment

The study was conducted between October 2021 and December 2023. The primary research setting was three large online groups on WeChat, a ubiquitous messaging and social media application in China that is deeply integrated into daily life and is often used for communication, medical consultation, and accessing health services. The characteristics of these groups are detailed in Table 1.

**Table 1:** Characteristics of the Wechat groups.

Item	Number	Region	Affiliation	Special Instructions
WeChat group one	500	China	CKD network service organization	The main purpose of the WeChat group is to sell drugs, health products, and food related to CKD treatment.
WeChat group two	289	Hubei province	Hospital	The WeChat group is used to strengthen the connection between the hospital and CKD patients and facilitate the organization of related activities.
WeChat group three	368	Wuhan	CKD patients independently establish	Through the introduction of acquaintances, the WeChat group was formed, mainly for CKD patients to communicate.

Access to this field was granted after a “gatekeeper,” a well-regarded patient advocate, introduced the primary researcher (W.X.) to the group administrators. Following a detailed explanation of the study’s objectives and ethical principles, the researcher was permitted to join the groups for observational purposes.

We used a combination of purposive and snowball sampling to recruit participants for in-depth interviews. The primary researcher first built rapport with community members over several months of passive observation and respectful interaction within the WeChat groups. This period of trust-building was crucial for gaining authentic access to the field. Recruitment was then conducted via private messages, inviting individuals to participate. The goal was to achieve maximum variation in the sample regarding age, gender, disease stage, and location to capture a wide breadth of experiences. Thirty individuals with CKD agreed to participate in offline semi-structured interviews. All participants were informed of their right to withdraw from the study at any time without consequence; none chose to do so after providing consent. The sociodemographic characteristics of the participants are presented in Table 2.

**Table 2:** Sociodemographic characteristics of participants.

Participants	Gender	Age	Location	Years with CKD	CKD Stage	Education	Occupation
P1	Female	29	Hubei	5	Stage 4	High School	Freelance
P2	Male	25	Hubei	3	Stage 2	University	Student
P3	Male	29	Henan	5	Stage 3	University	IT Professional
P4	Male	31	Hubei	6	Hemodialysis	High School	Unemployed
P5	Female	27	Hubei	4	Stage 4	Postgraduate	Teacher
P6	Male	26	Jiangxi	3	Stage 3	University	Sales
P7	Female	29	Hubei	5	Stage 5	University	Nurse
P8	Male	19	Hubei	2	Stage 2	High School	Sales
P9	Male	28	Hubei	4	Stage 3	University	Office Worker
P10	Male	30	Yunan	5	Transplant	Postgraduate	Manager
P11	Male	25	Hubei	3	Stage 2	University	Student
P12	Female	26	Hubei	5	Stage 1	University	Designer
P13	Male	22	Henan	2	Stage 1	University	Student
P14	Female	29	Hubei	4	Transplant	University	Unemployed
P15	Male	30	Hubei	7	Hemodialysis	University	Unemployed
P16	Male	28	Hubei	6	Stage 3	University	Office Worker
P17	Female	26	Jiangsu	3	Stage 3	High School	Freelance
P18	Female	25	Hubei	4	Stage 3	University	Student
P19	Male	29	Hubei	5	Stage 5	Postgraduate	Doctor
P20	Female	28	Hubei	3	Stage 3	University	Office Worker
P21	Male	27	Hubei	4	Stage 3	University	IT Professional
P22	Female	31	Hubei	8	Stage 5	University	Office Worker
P23	Male	29	Henan	5	Stage 5	High School	Manual Laborer
P24	Female	23	Hubei	1	Stage 2	University	Student
P25	Male	29	Hubei	5	Stage 3	University	Manual Laborer
P26	Male	19	Guangdong	1	Stage 1	High School	Manual Laborer
P27	Male	29	Hubei	7	Hemodialysis	University	Freelance
P28	Female	26	Hubei	3	Stage 1	University	Office Worker
P29	Male	59	Hubei	12	Hemodialysis	High School	Retired
P30	Female	27	Henan	4	Hemodialysis	University	Unemployed

### Data collection

One author (W. X., a male and experienced qualitative anthropologist) conducted all observations and interviews, and the other author (TH. A., a female and experienced physician) supervised these activities. Data were collected through two primary methods: (1) Participant Observation: Observations were conducted in two contexts: online within the WeChat groups and offline during patient-organized activities (e.g., dinners, parties, and sharing sessions). The researcher also conducted in-depth participant observations of five individuals’ daily lives to explore their family, work, and social interactions. All observations were documented in detailed field notes. (2) Semi-structured Interviews: Interviews were conducted as private, one-on-one interactions at a location chosen by the interviewee, with each session lasting 30–90 min. Most interviews were conducted once; after initial data analysis, brief follow-up phone calls were made to five participants to further clarify key issues. A semi-structured interview guide was developed for this study and pilot-tested with two individuals with CKD (not included in the final sample) to refine question clarity and flow. The guide was structured into three main sections. The first section explored the experience of illness and treatment, with questions such as “How long have you had kidney disease?”, “What do you think is causing your kidney disease?”, “How do you treat kidney disease?”, “When you think about having kidney disease, what are you most worried about for the future?”, and “Please tell me about some of the effects of kidney disease on your daily life.” The second section focused on social stigma, asking, “In your daily life, would you tell others that you have kidney disease?”, “How would other people react if they knew you had kidney disease?”, “Have you experienced any stigma since you developed kidney disease?”, “Can you please share your experiences with stigma in your life?”, and “Do you feel that stigma has impacted the progression of your kidney disease?” Finally, the third section addressed coping strategies, with questions including “How do you balance disclosure and concealment of information about kidney disease?”, “How do you feel about the stigma from the outside world?”, and “How do you deal with the stigma from the outside world?”

Interviews were audio-recorded and subsequently transcribed verbatim. Data collection continued until theoretical saturation was achieved, meaning no new data would alter our coding schema or the theories we devised to explain trends.

### Data analysis

We employed a thematic analysis approach, guided by the principles of focused ethnography. The analysis was an iterative process involving several stages. First, all interview transcripts and field notes were read repeatedly by two authors (W.X. and TH.A.) to gain a holistic understanding of the data. During this initial phase, the researchers independently performed open coding on a subset of transcripts, generating initial codes that closely reflected the participants’ language and experiences. Throughout this process, we recorded analytical and theoretical memos to capture our thought processes and data observations^38^. The NVivo software (version 12) was used to manage the data.

Next, the research team met regularly to compare codes, discuss interpretations, and develop a unified coding framework. This framework was then systematically applied to the entire dataset. Through a process of constant comparison, codes were grouped into larger, more abstract categories. These categories were then refined, merged, or split to form emergent themes that captured the core patterns of meaning related to stigma and coping strategies. Finally, these themes were organized into a coherent analytical narrative, supported by representative quotations from the data. To ensure confidentiality, all participants were assigned a unique identifier (P1 to P30), which is used throughout the presentation of the results.

### Trustworthiness of the findings

To ensure the trustworthiness of our findings, we employed several foundational strategies for rigor. The credibility of our interpretations was strengthened through data triangulation—synthesizing data from online discussions, field notes, and interviews—and the primary researcher’s prolonged engagement in the field for over two years. For analytical rigor, the two authors, with diverse backgrounds in anthropology and clinical nephrology, held regular peer debriefing sessions to resolve interpretive discrepancies. Furthermore, a comprehensive audit trail of raw data, memos, and coding schemes was maintained to ensure the dependability and confirmability of the research process.

To further validate our data and analysis, we centered participant involvement in the process. We returned interview summaries to five participants to confirm transcript accuracy. Subsequently, we conducted member checking with six participants by presenting our core analytical themes for their feedback; all affirmed that our interpretations resonated with their lived experiences, which greatly enhanced the credibility of our conclusions. Finally, we provide a “thick description” of the research context and participants throughout the manuscript, allowing readers to assess the transferability of the findings to other settings.

### Ethical considerations

The present study was approved by the Medical Ethics Committee, Union Hospital, Tongji Medical College, Huazhong University of Science and Technology (UHCT-IEC-SOP-016-03-01). All experiments were performed in accordance with relevant guidelines and regulations, including the Declaration of Helsinki. All participants were fully informed about the study’s purpose and procedures. They were assured of anonymity and the confidentiality of their data. Written informed consent was obtained from all 30 participants before conducting the offline semi-structured interviews.

## Results

### Stigmatization of CKD in China

Traditional medicine is widely used globally, with varying perceptions and understandings of the kidney across cultures, influencing prevention, treatment, and management of kidney disease^39^. In traditional Chinese medicine, the kidney is considered crucial. According to the renowned text “Difficult Classic (难经)”,

*There are two kidneys*,* but they are differ: the left is the kidney*,* and the right is the gate of life*,* home to various spirits and vital energies. For men*,* it stores sperm; for women*, *it is essential for childbearing. (*肾两者, 非皆肾也, 其左者为肾, 右者为命门。命门者, 诸神精之所舍, 原气之所系也, 男子以藏精, 女子以系胞*)*^40^.

This view shapes the Chinese perception of the kidney, leading to people with CKD stigmatization in China, manifested in four ways:

#### (1) stigmatization regarding the sexual lives of people with CKD

There is a belief that links kidney disease to sexual activity, suggesting that indiscriminate sexual behavior is a key cause of CKD. While clinically, significant sexual dysfunction is more commonly associated with advanced stages of CKD or individuals undergoing dialysis, our findings indicate that the social stigma linking kidney health to virility affects patients regardless of their disease stage. Such views lead to moral accusations against individuals with CKD, labeling their private lives as chaotic and promiscuous. This stigma often manifests first among peers, as a young male student recounted.

“*When I was first diagnosed*,* I thought I could talk to my friends about it. But instead of supporting me*,* they mocked me. They started making jokes about my personal life*,* saying I must have gotten sick because I had multiple girlfriends. It was unbelievably humiliating. Since then*,* I’ve learned to keep my mouth shut. I no longer tell anyone about my condition.*” (P2).

This judgmental attitude is not confined to friends but can also emerge from close confidants, creating profound emotional distress. Another young woman shared her experience:

“*The moment I was diagnosed*,* I felt utterly devastated and called my close friend. When she learned I had kidney disease*,* she asked if I had recently gotten a boyfriend and had been intimate. I roared that I hadn’t*,* and that my personal life was clean.*”(P5).

In some cases, the stigma originates from the closest family members, intertwining medical diagnosis with moral judgment:

*“Due to lower leg edema*,* my mother accompanied me to the hospital*,* where I was diagnosed with chronic nephritis. Upon receiving the diagnosis*,* my mother was momentarily shocked and inquired whether I had engaged in a romantic relationship involving sexual activity. I firmly denied this*,* but she remained unconvinced. Upon returning home*,* this issue led to persistent disputes*,* with my mother insisting on further medical tests to determine if I had contracted HIV. In her perception*,* kidney-related issues were strongly linked to sexual behavior.”*(P7).

#### (2) stigmatization of sexual capabilities

A prevalent belief in China holds that kidney damage impairs sexual function. This stigma particularly affects Chinese men with CKD, whose sexual abilities are frequently doubted. One participant described how this assumption played out in his professional life.

*“After my nephritis diagnosis*,* I did not inform my company. However*,* when I needed to file for medical insurance reimbursement*,* I submitted my medical records to the company. Soon*,* everyone at work knew about my condition. During company dinners*,* colleagues would order animal kidneys for me*,* claiming it would “nourish through similarity.” They even inquired about our private life from my wife*,* asking whether I still had sexual function. This was deeply embarrassing for both of us.”*(P10).

This focus on sexual function extends beyond the workplace and into casual social interactions, as another participant noted:

“*After my nephritis diagnosis*,* my husband and I felt it was not a significant issue*,* as it was only stage 1 and very mild. However*,* when our mutual friends learned about it*,* they asked whether we still had a sex life. I found this astonishing and initially explained to them that nephritis is unrelated to sexual activity. Nevertheless*,* in casual conversations*,* they frequently reminded me to reduce sexual activity*,* claiming my kidneys were already compromised.*”(P28).

#### (3) stigmatization related to fertility

There is a widespread belief that kidney damage compromises a woman’s fertility. This can have devastating consequences on major life events, such as marriage, as one female participant recounted.

*“My ex-boyfriend and I were preparing to get married. However*,* during a pre-marital health checkup*,* I was diagnosed with chronic kidney failure. The doctor said my condition was mild*,* but when my fiancé’s mother learned about it*,* she immediately demanded that he break up with me. She believed that my kidney condition would prevent me from having children*,* or that any children I might have would be unhealthy. As a result*,* the wedding was canceled. I don’t know if I can ever trust someone again or if I can ever marry.*”(P12).

For women who are already mothers, the stigma can manifest as doubts about their children’s paternity, as another participant shared:

“*I was diagnosed with chronic kidney disease at age 24*,* and a biopsy revealed it was stage 4. At the time*,* I was already married*,* and my daughter was born when I was 26. Some friends privately asked me if my daughter was biologically mine. I was furious and explained that kidney disease does not affect fertility.*”(P1).

#### (4) stigmatization of life status

Based on the traditional view of the kidney as the “gate of life,” many believe that kidney damage is irreversible and leads to premature death. This fear has intensified with the rising number of people with CKD in China. This stigma creates a burden of fear not only for the individual but also for their family, as a young university student explained.

“*I am 22 years old and currently a university student. During a physical examination last year*,* I was diagnosed with chronic nephritis*,* and a subsequent biopsy revealed it was stage 1. Initially*,* I was confident*,* believing that by adjusting my lifestyle and with advancements in medical science*,* my condition would not significantly impact my future quality of life. However*,* my mother frequently breaks down in tears*,* convinced that I will not live long and constantly worried about the tragedy of outliving her child.*”(P13).

For older individuals, this stigma can directly impact their professional lives and lead to social isolation, as a participant on hemodialysis shared:

*“I am 59 years old and work at the Health and Epidemic Prevention Control Center*,* originally planning to retire at 65. Prior to this*,* I had been undergoing hemodialysis treatment for eight years*,* scheduling it after work hours or during holidays. However*,* two years ago*,* China’s pandemic policies forced me to adjust my hemodialysis schedule. As a result*,* I had to disclose my condition to my supervisor*,* leading to workplace rumors that I was terminally ill and reliant on hemodialysis to survive. Subsequently*,* I frequently received condolence calls from colleagues. By the end of 2022*,* with the relaxation of pandemic measures*,* I had hoped to return to normalcy*,* but many colleagues still believed my condition was fatal. Ultimately*,* I stopped justifying my situation and sought early retirement to avoid further embarrassment.”*(P29).

Apart from these, there are also less typical stigmas, such as advocating that CKD is contagious, physical distancing from people with CKD, and psychological rejection of them.

### Strategies for coping with stigmatization by Chinese people with CKD

Despite facing stigmatization, Chinese people with CKD aren’t entirely passive; instead, they actively use various resources and adopt a dualistic strategy to foster a supportive micro-environment. This approach, which many participants described as “huddling for warmth (抱团取暖),” involves a careful balance of external concealment and internal community building to manage their social identity and emotional needs.

#### Concealment as a protective shield

The most common external strategy is the deliberate concealment of their diagnosis from the wider social circle, including colleagues and friends. This acts as a protective shield against potential misunderstanding, judgment, and discrimination. Participants explained this was not born of shame, but a pragmatic choice to avoid unnecessary psychological stress. One participant articulated this rationale:

“*It’s better not to disclose your CKD; misunderstandings are widespread*,* and clarifying them is often futile. people with CKD should maintain a positive attitude. Revealing our condition leads to being viewed differently. Sharing only adds to our psychological stress without any benefit. To preserve good relationships*,* I’ve kept my condition from colleagues and friends. When dining out*,* I I adhere to the dietary guidelines recommended by my doctor. When they recommend food that is unsuitable for my condition*,* I simply say that I don’t like those items*,* rather than telling them the truth.”* (P22).

This concealment can also take the form of providing alternative explanations for their health-related behaviors. Another participant demonstrated this adaptive strategy:

*“I opted not to disclose my CKD*,* instead saying I had familial high blood pressure requiring regular doctor visits for monitoring. Hearing about high blood pressure*,* they didn’t see it as a major issue but encouraged timely medication. If I tell them I have CKD*,* there will be a lot of criticism*,* which is inevitable in Chinese society. I didn’t want to get into trouble*,* so I had to use the strategy of deception.”* (P11).

#### Community Building for internal support

While concealing their condition from the outside world, participants simultaneously turn inward to build strong, supportive communities with fellow “kidney friends (肾友)”. Digital platforms, particularly WeChat, have become vital spaces for this community formation. Here, in the relative anonymity and safety of a shared-identity group, individuals can shed their protective shields. Our observations show that these online groups evolve from simple information-sharing platforms into close-knit communities where members find emotional support, exchange practical advice, and cultivate kinship-like bonds, often transcending social differences. One participant, who became an administrator of a CKD WeChat group, described the profound impact of this community:

“*The CKD WeChat groups offer a communication and interaction platform for us*,* kidney friends. Being patients*,* we quickly develop a strong emotional bond. In the WeChat group*,* although we are strangers*,* we become like family*,* readily assisting anyone in need. This sense of community boosts confidence among people with CKD and aids in overcoming challenges. As a result*,* our WeChat group thrives*,* engaging in lively discussions on a wide array of topics*,* extending beyond just CKD. CKD has undoubtedly altered our lives*,* forcing us to adapt. Yet*,* the formation of the CKD WeChat group has transformed our lives anew*,* unveiling the potential for improved living.”* (P25).

For patients who become more isolated due to their condition, these online communities can become their primary social lifeline, as explained by one patient who has been unemployed since undergoing a kidney transplant:

*“After my kidney transplant*,* I didn’t go back to work. My social circle is very limited*,* and I don’t want to talk about my condition with acquaintances to avoid awkwardness. However*,* I spend a lot of time chatting in the WeChat groups every day; this has become my main form of social interaction. Besides sharing information about our conditions*,* we also share stories from our daily lives. More importantly*,* everyone encourages each other*,* which gives me immense psychological comfort. Perhaps because we are all kidney disease patients*,* we have more in common and can better understand each other’s situation.” (P14)*.

This dual strategy of “huddling for warmth”—distancing from external judgment while embracing internal solidarity—showcases the resilience and agency of Chinese people with CKD. It is a sophisticated, self-developed mechanism for preserving well-being within a challenging socio-cultural environment.

## Discussions

Although studies have shown that better environmental protection, inclusion of CKD in the national public health surveillance program, and control of common CKD comorbidities have reduced the burden of disease for people with CKD in China^41^the social and cultural environment in which they live has not been fundamentally changed. 

Confronted with entrenched social prejudices and cultural stereotypes, Chinese people with CKD recognize the long-term nature of their stigma and thus adapt their survival strategies accordingly. Through the way of “huddling for warmth”, Chinese people with CKD bridge the gap between external stigma and internal emotional needs, fostering better living conditions and societal integration. This underscores the resilience and agency of Chinese people with CKD.

This study reminds us that research on the situation of people with CKD requires a multidisciplinary approach. Alongside nephrology and public health, social science interventions are crucial for understanding CKD’s local experiences and its socio-cultural associations^42^. Additionally, comprehending socio-cultural impacts on CKD will facilitate global mapping and enhance solutions to the worldwide surge in CKD.

This study has some limitations. The data collection period (October 2021 to December 2023) coincided with the COVID-19 pandemic. While the pandemic’s impact is partially reflected in our data (e.g., disruptions to dialysis schedules experienced by P29), a focused analysis of its specific effects on stigma was beyond this study’s scope and warrants future research. Furthermore, our cohort has a relatively young average age. This may have influenced the findings, leading to a greater emphasis on stigma related to career, dating, and marriage, and a higher proficiency in using online platforms for community support. The experiences of older adults with CKD may differ, representing another important avenue for future investigation.

## Conclusions

Influenced by traditional Chinese medicine, the Chinese link the kidney to sex, fertility, and life. This concept shapes the Chinese perspective on CKD, resulting in stigma and discriminatory practices against people with the condition.

In the face of strong traditional beliefs, it is difficult for Chinese people with CKD to completely reverse society’s misconceptions about the kidney. Consequently, they adopt a flexible survival strategy, concealing their CKD status publicly while fostering a community identity privately. By “huddling for warmth,” Chinese people with CKD merge individual with societal, internal with external, and traditional with modern aspects, highlighting their resilience and agency.

From a public health perspective, these findings are highly relevant as they demonstrate that cultural stigma is a significant social determinant of health, acting as a barrier to well-being that can negatively impact mental health and treatment adherence. Therefore, designing and implementing culturally sensitive public health campaigns and provider education programs to combat this stigma is not merely a social issue but a crucial component of comprehensive and effective CKD management in China .

However, the vast number of people with CKD in China cannot solely depend on individual or group efforts to address their challenges. The Chinese government, medical institutions, and the public must focus on this group’s needs^35^from health legislation to population-based programs, creating a healthcare infrastructure that reflects medical needs and humanistic values^43^.

Additionally, combating the stigma encountered by Chinese people with CKD necessitates integrating qualitative research with public health interventions. Qualitative research plays a crucial role in developing interventions, assessing their feasibility, acceptability, appropriateness, and determining the best evaluation strategies^44^. It is only by returning to the daily life of people with CKD that we can understand the stigmatizing discourses and practices they face and the socio-cultural context behind the underlying stigmatizing discourses, and thus propose tailored interventions to address them.

## Electronic supplementary material

Below is the link to the electronic supplementary material.

Supplementary Material 1

## Data Availability

The datasets generated and/or analyzed during the current study are not publicly available due to the sensitive nature of the interviewed population. To protect the rights and interests of the participants, we have decided not to share the data. However, the datasets are available from the corresponding author on reasonable request.
